# Impact of Beam Deflection Geometry on the Surface Architecture and Mechanical Properties of Electron-Beam-Modified TC4 Titanium Alloy

**DOI:** 10.3390/ma16155237

**Published:** 2023-07-26

**Authors:** Maria Ormanova, Borislav Stoyanov, Nikolay Nedyalkov, Stefan Valkov

**Affiliations:** 1 Academician Emil Djakov Institute of Electronics–Bulgarian Academy of Sciences, 72 Tsarigradsko Chaussee Blvd, 1784 Sofia, Bulgaria; nned@ie.bas.bg (N.N.); stsvalkov@gmail.com (S.V.); 2 Department of Industrial Desing and Textile Engineering, Technical University of Gabrovo, 4 H. Dimitar Srt, 5300 Gabrovo, Bulgaria; stoyanov_b@mail.bg; 3 Department of Mathematics, Informatics and Natural Sciences, Technical University of Gabrovo, 4 H. Dimitar Srt, 5300 Gabrovo, Bulgaria

**Keywords:** electron-beam modification, TC4 titanium alloy, beam deflection geometry, phase composition, structure, surface architecture, coefficient of friction

## Abstract

This paper aims to investigate the impact of beam deflection geometry on the structure, surface architecture, and friction coefficient of electron-beam-modified TC4 titanium alloys. During the experiments, the electron beam was deflected in the form of different scanning geometries, namely linear, circular, and matrix. The structure of the treated specimens was investigated in terms of their phase composition by employing X-ray diffraction experiments. The microstructure was studied by scanning electron microscopy (SEM). The surface architecture was examined by atomic force microscopy (AFM). The friction coefficient was studied by a mechanical wear test. It was found that the linear and circular deflection geometries lead to a transformation of the phase composition, from double-phase α + β to α’ martensitic structure. The application of a linear manner of scanning leads to a residual amount of beta phase. The use of a matrix does not tend to structural changes on the surface of the TC4 alloy. In the case of linear geometry, the thickness of the modified zone is more than 800 μm while, in the case of EBSM using circular scanning, the thickness is about 160 μm. The electron-beam surface modification leads to a decrease in the surface roughness to about 27 nm in EBSM with linear deflection geometry and 31 nm in circular deflection geometry, compared to that of the pure TC4 substrate (about 160 nm). The electron-beam surface modification of the TC4 alloy leads to a decrease in the coefficient of friction (COF), with the lowest COF values obtained in the case of linear deflection geometry (0.32). The results obtained in this study show that beam deflection geometry has a significant effect on the surface roughness and friction coefficient of the treated surfaces. It was found that the application of a linear manner of scanning leads to the formation of a surface with the lowest roughness and friction coefficient.

## 1. Introduction

Titanium-based alloys have been widely used for the production of orthopedic implants and prostheses [1,2,3], as well as for orthodontic archwires and dental instruments [4,5], due to their significant number of exceptional properties (good corrosion resistance, better biocompatibility, and higher specific strength). However, when placing implants in the human body, a number of adverse reactions may occur (fracture of the implant, deterioration of adhesion, release of metal ions, etc.), leading to a new intervention or the course of acute inflammatory processes [6]. Therefore, the Ti-based alloys used in implantology have to possess suitable functional properties, such as a low Young’s modulus, low coefficient of friction, etc. The modulus of elasticity has to be close to that of human bones. [7,8]. In this case, stresses at the implant–bone interface could be significantly reduced and the longevity of placed implants increased.

The improvement of the surface properties of implant materials could be achieved by different surface modification methods [9,10,11,12]. In general, methods for surface modification of implant materials can be classified as physical, chemical, and mechanical [13,14,15]. These methods and techniques can be used both individually [16,17,18] and in combination [19,20,21]. It is essential to choose a suitable technique for the modification of surfaces of implants depending on their specific applications [22,23,24].

Surface treatments of metals and alloys by a flux of accelerated electrons comprise a commonly used technology used to improve the performance characteristics of materials [23,25,26,27]. These techniques are considered very reproducible, low cost, and have very low process times [28]. The mechanism of electron-beam surface modification (EBSM) is based on the transformation of the kinetic energy of electrons into heat, which is accompanied by a distribution of heat from the surface to the bulk [28,29,30]. High heating and cooling rates lead to phase and structural changes that significantly improve the surface properties of metals and alloys [31,32]. The electron-beam surface treatment can be performed via different scanning trajectories of the electron beam, namely linear, circular, matrix, etc. [33]. In this way, precise control of the heating and cooling rates and, therefore, the obtained structure and functional properties of the material can be achieved.

The authors of [32] have discussed the effect of low-energy high-current pulsed electron-beam (LEHCPEB) surface treatment on the structure and properties of a near-α Ti alloy. The results showed that the LEHCPEB process leads to phase and structural transformations, leading to a significant enhancement in the surface hardness and corrosion properties of the titanium alloy.

Gao [34] has studied the effect of electron-beam surface treatment on the structure and properties of titanium alloys. The work-pieces were modified in a pulsed mode and experiments were realized under different pulsed energy densities, durations of pulses, and numbers of pulses. It was found that the hardness was improved due to the higher number of dislocations caused by heat stresses. Similarly, Gao [35] investigated the pulsed electron-beam modification of TA15 titanium alloy where the kinetic energy was in the range of 10–40 keV and the results showed that the microstructure was significantly refined, leading to an increase in the microhardness. In Refs. [36,37] the electron-beam technique was used to design a specific surface (cp-Ti and Ti6Al4V alloy) to control the effect of contact guidance and limit microfouling. The results showed a change in crystallographic structure and microstructure; moreover, a significant reduction in bacterial adhesion was observed in the EB-structured samples. The relationship between the microstructure of titanium alloys and antiadhesive activity against bacteria opens a new strategy for the development of innovative antiadhesive/antibacterial metallic materials [36,37]. Our previous investigations [38] were based on the study of the change in the phase composition and microstructure of Ti6Al4V alloy surfaces after electron-beam surface treatment. During the experiment, the electron-beam current was in the range of 20 mA to 25 mA, and a circular manner of scanning was applied. The results showed a change in the phase composition and microstructure of the surface of the treated samples. A typical martensitic transformation for the electron-beam-processed titanium alloys was observed. The structural changes that occurred had a significant impact on the improvement of the surface hardness of the titanium alloy samples.

It is obvious that the use of electron-beam surface modification is very suitable for the improvement of the surface properties of a number of metals and alloys. However, investigations on surface modification by a more complex manner of scanning, as well as those on the influence of the beam deflection geometry on the functional properties of Ti-based materials, are currently lacking in the scientific literature. As already mentioned, the use of different manners of scanning, defined by the geometry of beam deflection, leads to different thermal distributions from the surface to the bulk and, therefore, different thermal cycling gradients during the electron-beam surface modification procedure. This leads to different structures and corresponding functional properties. Thus, the aim of the present work is to study the impact of beam deflection geometry on the surface architecture and mechanical properties of an electron-beam-modified TC4 titanium alloy. The presented results here provide new information regarding structure and property modifications associated with electron-beam processing when different geometries of beam deflection are considered. This study could be a basis for the design and development of advanced techniques for material surface structuring with tunable properties.

## 2. Materials and Methods

The TC4 titanium alloy samples used in this study had a cylindrical shape with diameter of 30 mm and thickness of 4 mm. The chemical composition of the samples is shown in Table 1.

**Figure 1:**
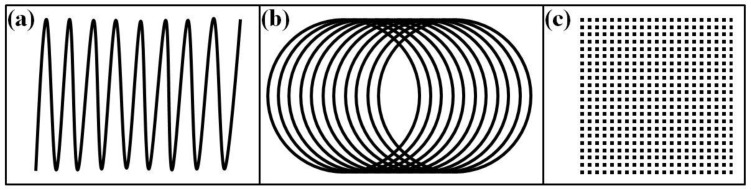
Schematical representation of the beam deflection geometries used: (**a**) line; (**b**) circle; (**c**) matrix.

The surfaces of the samples were mechanically polished (grinding papers of 320 to 1000) and ultrasonically cleaned in water. The surface modification was conducted by means of a scanning electron beam using an Evobeam Cube 400 machine (Evobeam GmbH, Nieder-Olm, Germany). The following technological conditions were applied: accelerating voltage—60 kV; electron-beam current—25 mA; scanning frequency—1 kHz; speed of sample’s motion—5 mm/s. The beam deflection geometry was in the form of a line (sample 1), a circle (sample 2), and a matrix (sample 3). All experiments were realized under the same technological conditions with an oscillation amplitude of 16 mm. The selection of the discussed technological conditions was based on systematical optimization and the parameters presented above were considered as the most representative. It was established that for much lower values of electron-beam current, as well as for higher values of speed of movement and scanning frequency, the surface temperature became insufficient and a modification of the structure and properties was not achieved. At significantly higher values of beam current, as well as lower values of speed of movement and scanning frequency, very strong modifications of the surface topography occurred. Also, the melting processes became predominant which was not considered a desirable effect. The geometries of the beam deflection are shown in Figure 1. These are available in most commercial electron-beam systems and their use expresses a variety of conditions wherein different heating dynamics can be realized. Several experiments were realized under these technological conditions and the corresponding results were identical, as their deviation was in the range of 10%, confirming their reproducibility.

The phase composition of the specimens was examined by X-ray diffraction (XRD, Philips PW1050, Amsterdam, The Netherlands) with Cu Kα radiation (1.54 Å). The measurements were recorded within the range of 30° to 80° at a 2-theta scale.

The microstructure and chemical composition of the obtained samples were investigated by scanning electron microscopy (SEM-LYRA I XMU (Tescan), Brno, Czech Republic). During the measurements, secondary and back-scattered electron modes were used.

The surface topography and roughness of the obtained samples were examined via Atomic Force Microscopy (AFM) (Asylum Research, Oxford Instruments) with a Si-AC160TS-R3 silicon tip. During the experiments, a silicon tip with a radius of 10 nm was used to scan an area of 20 × 20 μm.

The coefficient of friction was determined by a mechanical wear test (ball-on-flat) using a micro-tribotester (UMT-2, Bruker, CA, USA) with a sliding ball coated with Cr. The measurements were carried out for a time of testing of 900 s at a load of 5 N. The tests were conducted in the air and at room temperature.

## 3. Results and Discussion

**Figure 2:**
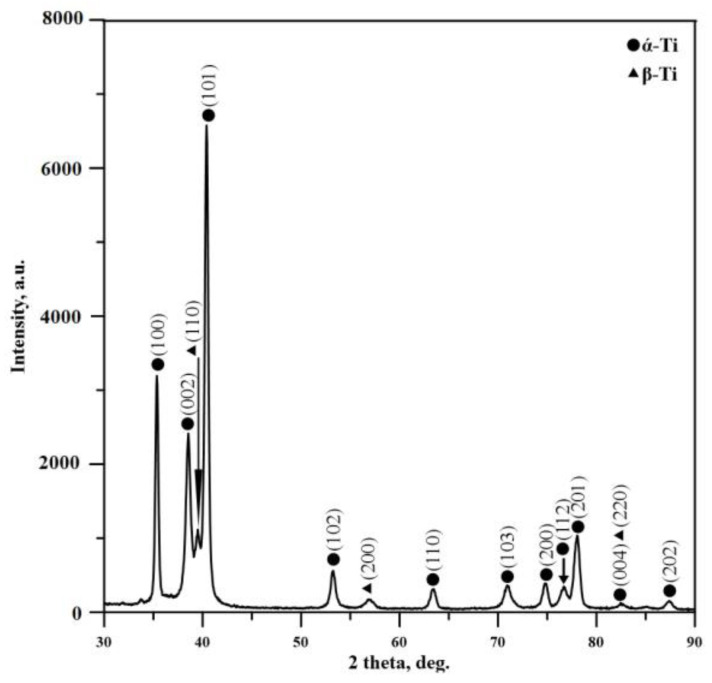
X-ray diffraction pattern of the pure TC4 titanium alloy.

Microstructural studies of pure TC4 alloy are presented in detail in our previous works [19,38] in the form of a double-phase structure of α and β phases, which is typical for TC4. This is the typical microstructure for such kinds of alloys. It is known that the beta phase is the high-temperature modification of the titanium and is stable at a temperature higher than 1153 K. However, in Ti–Al–V alloys, the mentioned phase appears due to the existence of the vanadium element. It is known that V plays a role of a beta-stabilizing element and, therefore, the appearance of this structure in the alloy at room temperature is attributed to the existence of vanadium atoms [39]. These statements were confirmed by XRD experiments. Figure 2 presents the experimentally obtained XRD pattern of TC4 titanium alloy before electron-beam surface modification. The identification of the phase composition was performed according to the International Centre for Diffraction Data (ICDD) database, PDF # 44-1294 for α-Ti and PDF # 44-1288 for β-Ti. The phase composition of the untreated TC4 alloy is in the form of a biphasic structure of α and β phases, as confirmed by the presence of α-Ti and β-Ti diffraction maxima. It should be noted that peaks of the beta phase are shifted in comparison with their position available in the JCPDS crystallographic database. The data available in this database were obtained concerning the high-temperature nature of the discussed phase. However, the presence of the second-phase bcc beta structure in this case was due to the existence of beta-stabilizing elements (vanadium in the present case) and its lattice parameter and volume strongly depend on the atomic radii of V, leading to a different peak position of the discussed diffraction maxima.

**Figure 3:**
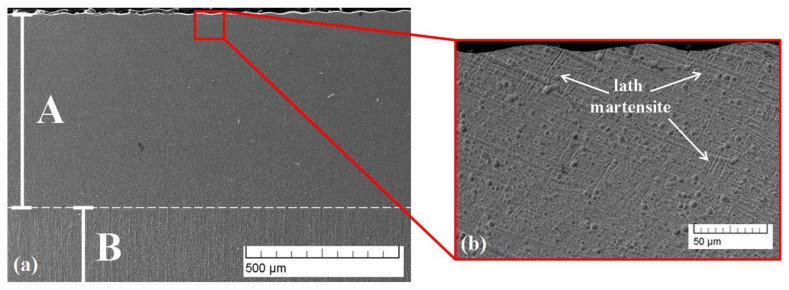
Cross-sectional SEM micrographs of the microstructure of sample 1 (line): (**a**) small magnification; (**b**) higher magnification.

Cross-sectional SEM micrographs of sample 1 modified by a linear deflection geometry are shown in Figure 3. The modified area is marked as area A, and the pure TC4 substrate is noted as B (Figure 3a). The thickness of area A is about 816 μm. The martensitic structure within the treated zone is clearly distinguishable (Figure 3b). Also, during the treatment process, a high-temperature gradient exists, leading to the finer structure of the modified area.

**Figure 4:**
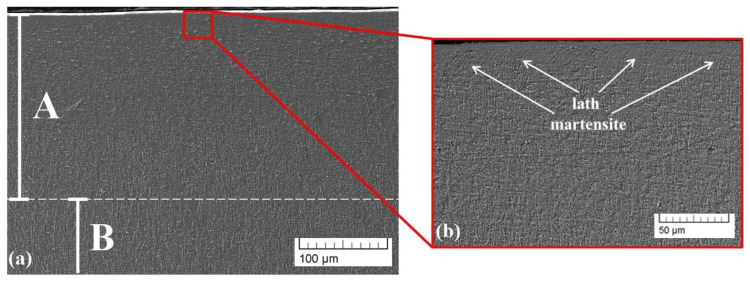
Cross-sectional SEM images of the microstructure of sample 2 (circle): (**a**) small magnification; (**b**) higher magnification.

The experimentally obtained cross-sectional SEM images of the microstructure of sample 2 (circular geometry) are presented in Figure 4. The treated zone is marked as zone A, and the base material is noted as B (Figure 4a). The thickness of the area A is about 162 μm. Compared to sample 1 (linear geometry), the electron-beam modification with circular geometry leads to the formation of a modified zone with a significantly smaller thickness. Therefore, the beam deflection geometry can significantly affect the thickness of the modified area. According to the authors of [28,33], in electron-beam surface treatment processes, a thermal distribution from the surface to the depth of the specimen is formed and strongly depends on the geometry and shape of the scanning figure, defined by the deflection of the electron beam. In the case of the circular manner of scanning, the scanned area is much larger in comparison with the case of linear deflection and, therefore, the resultant surface and in-depth temperatures have to be lower. Moreover, the dimension of the circle is larger than the line, meaning that the overall path of the electron beam is longer. At the same time, the frequency of scanning is the same and, therefore, the scanning of the beam via each deflection mode is realized identically. This means that in the case of the larger scanning figure (i.e., circle), the path of the electron beam is longer and in order for one period of scanning to take the same amount of time to be realized, like in the case of the linear approach, the speed of the beam movement over the surface of the treated specimen must be higher. This higher velocity of the beam for the circular-approach surface treatment is characterized by a lower heat input, which could be another possible reason for the thinner treated area. According to our previous investigation [38], electron-beam surface treatment with a circular geometry of beam deflection using a smaller amplitude of scanning (10 mm) and very similar technological conditions and parameters led to the formation of a deeper treated zone of about 500 μm. In that case, the path of the electron beam is comparable with those of the linear geometry of beam deflection used in the present study, leading to a similar depth of treated zones in both cases. A martensitic microstructure is again visible on the surface of the considered sample. However, in the present case, it is obvious that the microstructure of the specimen processed by the circular beam deflection is finer in comparison with that treated by a linear manner of scanning. This could be attributed to the higher thermal cycling gradient in the case of circular-geometry scanning. As already mentioned, in the case of a linear deflection of the beam, the temperature is higher and the convective mixing processes within the treated zone become predominant, leading to a decrease in the cooling rate and, therefore, a coarser microstructure.

**Figure 5:**
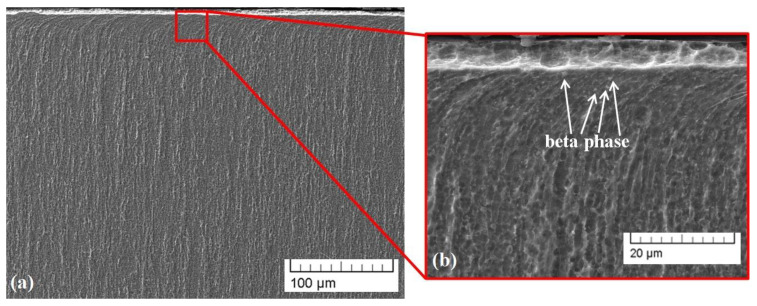
Cross-sectional SEM image of the microstructure of sample 3 (matrix): (**a**) small magnification; (**b**) higher magnification.

Figure 5 shows a cross-sectional SEM micrograph of the structure of electron-beam-modified sample 3 with matrix deflection geometry. Figure 5a shows a lower-magnification micrograph, while Figure 5b exhibits a higher-magnification image. The results obtained show that the electron-beam treatment procedure with beam deflection in the form of a matrix does not significantly influence the structure of the specimen. It is obvious that the microstructure is in the form of a double-phase structure of α + β Ti, which matches that of the initial specimen, i.e., before the treatment process. Furthermore, no significant refinement of the microstructure can be observed, which is in contrast with the previously considered specimens. As already mentioned, due to the nature of charged particles, the electrons can deflect from the normal axis via electromagnetic fields formed by so-called deflecting coils, leading to treatment and modification using different scanning figures, such as linear, circular, matrix, etc. [33]. The electron beam in the matrix geometry is split into a number of sub-beams (forming the structure presented in Figure 1c). In this case, the overall power of the incident electron beam is a sum of the powers of the sub-beams. Therefore, in the case of electron-beam surface treatment using a beam deflection in the form of a matrix, each spot has low power Ps (P_s_ = P_tot_/n, where n is the number of spots in the matrix). It is lower compared to the linear and circular cases where the electron beam’s total power was focused on the top of the specimens. Therefore, the temperature over the scanned zone in the case of electron-beam surface treatment with beam deflection in the form of a matrix is much lower and is not sufficient to modify the surface. Therefore, this specimen is not considered further.

**Figure 6:**
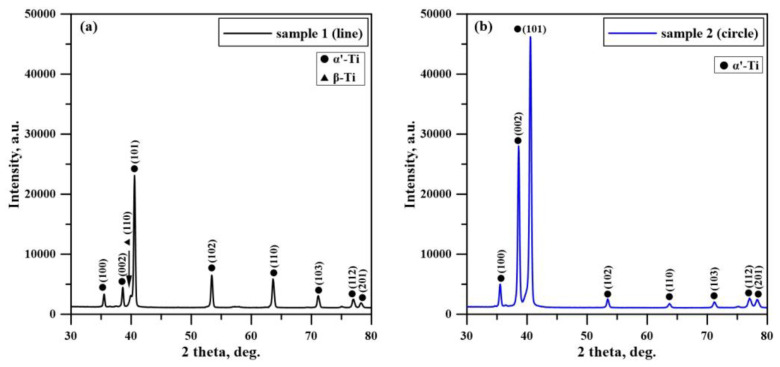
XRD patterns of the electron-beam-modified TC4 alloy: (**a**) sample 1; (**b**) sample 2.

X-ray diffraction patterns of the electron-beam-modified TC4 samples with different beam deflection geometries are shown in Figure 6. The experimentally obtained diffractograms of the two electron-beam-treated specimens (linear and circular geometry of beam deflection) exhibit diffraction peaks of α’ martensitic phase which is characterized by a hexagonal close-packed (hcp) structure. The diffraction maximum corresponding to the (101) crystallographic plane of the beta phase becomes negligible in both considered cases of surface modification. This could be attributed to the formation of the discussed martensitic structure. The formation of martensite is a result of the very high cooling rates that accompany the electron-beam surface modification process [28,33]. It is known that the α’ martensite is formed after rapid cooling from the field of β phase, where the high-temperature bcc structure undergoes a phase transition into a hcp martensitic structure. The electron-beam surface treatment process is characterized by very high cooling rates of 10^4^–10^5^ K/s. Therefore, such phase changes are typical for α + β titanium alloys, which are treated via high energy fluxes [40]. Such transformations from α + β to α’ martensitic structure during electron-beam surface processing of TC4 alloy are also observed by Ormanova et al. [38], Petrov et al. [39], and Nikolova, et al. [41]. Also, it should be mentioned that the intensities of the peaks belonging to the martensitic phase are changed significantly depending on the different geometries of beam deflection during the treatment procedure. Also, it is well visible that the ratios between the diffraction maxima corresponding to α’ martensitic phase strongly depend on the applied geometry of beam deflection. The peak intensity belonging to the (002) crystallographic plane is comparable with that of (100) in the case of a linear manner of scanning, while in the case of circular geometry, the intensity of (002) is much higher. It is important to note that this trend is completely opposite from the case of untreated TC4 alloy where the intensity of the (100) peak is higher than that of the (002) peak. Also, it is well visible that the ratio between (002) and (101) peaks is much smaller in the specimen modified by a circular approach, as compared to the linear one. At the same time, the intensities of the diffraction maxima belonging to (102), (110), and (103) crystallographic planes for the specimen processed by a circular manner of scanning are much lower than the intensities of the same peaks of the sample modified using linear geometry. All these features could be attributed to reorientations in the micro-volumes concerning the different geometries of beam deflection and cooling rates, which is typical for electron-beam treatment procedures due to the highly non-equilibrium conditions [39].

**Figure 7:**
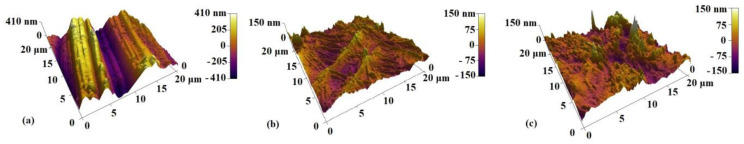
Three-dimensional (3D) AFM micrographs of the surface architecture of the samples: (**a**) base (pure TC4 alloy); (**b**) sample 1 (line); (**c**) sample 2 (circle).

Figure 7 presents the 3D AFM micrographs of the surface architecture of the pure TC4 titanium alloy (Figure 7a), namely sample 1, modified with a linear geometry (Figure 7b), and sample 2, modified with a circular geometry (Figure 7c). The modification process using a scanning electron beam has a significant influence on the surface topography and architecture of the examined samples. The untreated titanium sample exhibits a rough surface (Figure 7a). This indicates residual roughness despite the initial mechanical polishing with different grinding papers. On the other hand, sample 1 possesses a wave-like topography, with relatively homogeneously distributed surface formations (Figure 7b). During the EBSM process with a scanning electron beam, the molten material from the peaks flows down and fills the valleys. Thus, an almost flat surface is formed. Considering the surface architecture of the specimen treated with circular-geometry beam deflection, some peak-like formations on the flattened surface can be seen. In this case, the material stays in a molten state for a longer time due to the overlap of the beam trajectory, compared to the case of linear geometry. The process of the formation of the peaks may be related to the evaporation of the TC4 material and subsequent condensation of the vapors, despite the low surface temperature during treatment using the circular manner of scanning, which could be the reason for the formation of the discussed peak-like formations [42].

The results for the surface roughness of the studied samples are summarized in Table 2. Electron-beam surface modification can significantly affect the resultant surface architecture in two ways [43]. In the case of very high roughness, EBSM results in a decrease in the surface roughness as the molten peaks fill the valleys. On the other hand, the roughness may increase due to the formation of craters on the surface of the modified samples. In our particular case, the presence of residual roughness (Figure 7a) even after mechanical polishing is a prerequisite for a reduction in surface roughness after the EBSM process. This fact is confirmed by the drastic decrease in the discussed parameter to about 27 nm in EBSM with linear deflection geometry (sample 1) and to 31 nm in EBSM with circular deflection geometry (sample 2), compared to that of the initial TC4 substrate (about 166 nm). The formation of the discussed peak-like formations in the case of the circular manner of scanning is likely the reason for the slightly higher surface roughness than that of the sample treated with linear-geometry beam deflection. 

It should be noted that the results obtained on surface roughness are of significant importance in the field of modern biomedicine, mostly in implant manufacturing. It is known from the literature that the surface nanostructuring of biomaterials has a direct impact on the cellular response by increasing protein adsorption, bone cell migration, and osseointegration [44,45]. On the other hand, a higher roughness corresponds to a larger contact area, and this can stimulate bone regeneration and binding to bone tissue [46].

**Figure 8:**
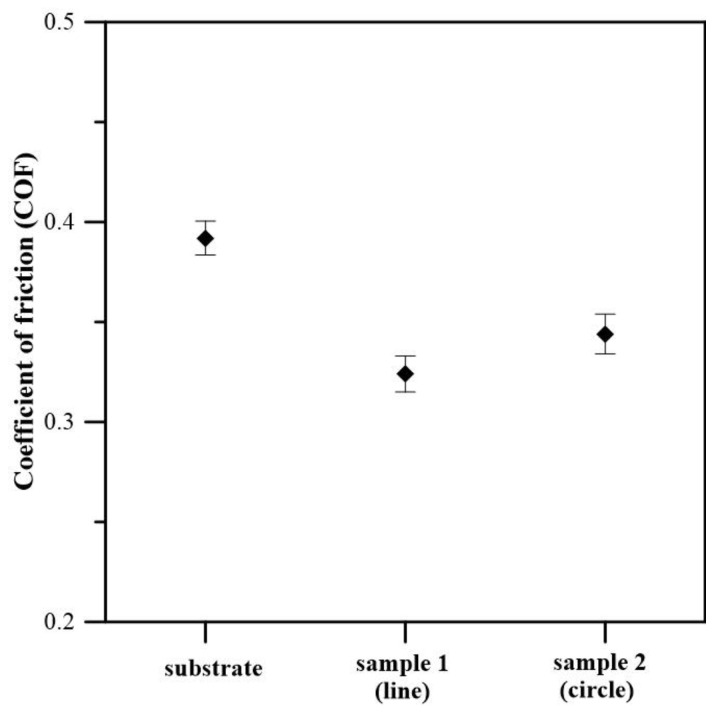
The average coefficient of friction of the TC4 substrate and electron-beam-modified TC4 samples.

The coefficient of friction (COF) of the pure TC4 alloy and the electron-beam-modified titanium samples was investigated by a mechanical wear test (ball-on-flat). The obtained results are shown in Figure 8 and are summarized in Table 2. The average value of the COF of the TC4 substrate is about 0.39, the COF of sample 1 (modified with linear deflection geometry) is about 0.32, and the COF of sample 2 (EBSM with circular deflection geometry) is about 0.34. The results show that surface modification with a continuous electron beam leads to a reduction in the coefficient of friction, with the lowest COF values obtained in the case of linear deflection geometry (0.32). Therefore, the friction coefficient of the modified surfaces is improved due to two different reasons. As already mentioned, the application of the electron-beam surface modification procedure leads to a transformation from α + β to α’ martensitic structure in both considered cases [33,38,39]. The latter is characterized by much better wear properties, which could be a reason for the decrease in the COF. On the other hand, the surface roughness also has a significant influence on the friction properties [47]. As was already established, the application of surface modification using a scanning electron beam tends to a decrease in the surface roughness and, therefore, to the friction coefficient. When the investigated surface is smooth, the main wear mechanism is abrasive wear, leading to relatively low values of the friction coefficient [48]. In the case of linear deflection geometry, the surface roughness is the lowest (about 27 nm), which corresponds to a low coefficient of friction (0.32), as a result of abrasive wear. The coefficient of friction of sample 2 (0.34) modified with a circular deflection geometry is also lower than that of the untreated specimen (0.39). This means that the wear mechanism in this case is also abrasive. In general, the COF of the electron-beam-modified TC4 alloy is lower than that of the initial TC4 alloy, indicating that EBSM can contribute to a reduction in the friction coefficient and improvement of the wear properties. This makes EBSM technology promising for enhancing the performance of implant materials, where lower friction coefficients and improved wear properties are of major importance. The authors of [49] have studied the coefficient of friction of different coatings (TiN, TiCN, and TiAlN) deposited on Ti6Al4V alloy for biomedical applications, and the results showed that the COF is in the range of 0.5 to 0.7 and concluded that the surface roughness is a major factor influencing COF values. The results obtained in this study are comparable with those published by the authors of [23], wherein Ti substrates were modified by the formation of surface alloys in a system of Ti–Ta.

In this study, results of electron-beam surface modification of TC4 alloy are presented. The impact of beam deflection geometry during the EBSM process on the phase composition, microstructure, surface architecture, and coefficient of friction of the modified samples was investigated. The results show that the discussed parameter (beam deflection geometry) influences the surface topography and roughness of the specimens.

This study concerns the electron-beam surface modification of TC4 titanium alloy which was realized using a deflection of an electron beam in the form of a line, circle, and matrix. The results show that the application of a linear manner of scanning leads to the deepest modified zone, while the use of a circular manner of scanning tends to the formation of a modified zone with a significantly smaller thickness. It should be noted that the rate of modification using linear and circular approaches of scanning is different due to the different dimensions of both figures. In the case of the circular geometry, the modified zone is thinner because of the larger dimension of the scanning figure, which results in a lower deposited energy density. The application of a beam deflection in the form of a matrix does not tend to the formation of a modified area using the present technological conditions. However, the question remains open regarding the influence of technological conditions and parameters on the structure and properties of electron-beam-modified metals and alloys using beam deflection in the form of a matrix. This is of major importance since it will provide new knowledge on the structure formation and corresponding functional properties of electron-beam surface modification technologies employing more complex manners of scanning.

The results for the microstructure obtained by both approaches of electron-beam treatment (i.e., using linear and circular manners of scanning) show that a fine martensitic structure was obtained in the two cases. During the experiments, the kinetic energy of the electrons is transferred into heat and a thermal distribution from the surface to the bulk is formed, where the cooling rate is very high (10^4^–10^5^ K/s), leading to the formation of significantly finer structure. As already mentioned, the temperature in the case of the linear manner of scanning is higher than that of the circular one, leading to a deeper modified zone. Considering the treatment using a circular manner of scanning, the decrease in the amplitude of scanning (i.e., the decrease in the diameter of the circle) leads to a rise in the surface temperature and a deeper treated zone. At the same time, the use of a circular approach of electron-beam modification leads to overlap of the trajectory of the beam (see Figure 1), leading to an increase in the maximal temperature and a lower cooling rate in comparison with the linear approach of surface modification and, therefore, coarser microstructure and worse functional properties are obtained. These statements are in agreement with those published in Ref. [33], where it is stated that a linear manner of scanning is much more appropriate for surface modification based on martensitic transformation. It should, however, be mentioned that careful choice of the processing conditions and the beam scanning geometry may result in a wide variety of material surface modifications since, as it is mentioned above, these define the temperature evolution of material which is the crucial parameter for phase transformations.

It was demonstrated the surface roughness is significantly lower in the case of the electron-beam modified TC4 alloy with linear deflection geometry than the initial TC4 substrate. It reached values of 26.70 nm, which is about six times lower in comparison with the untreated titanium alloy. The surface roughness values under circular-geometry beam deflection are 31.41 nm, which is about five times lower than that of the untreated alloy. As mentioned above, the surface roughness of biomaterials on macro-, micro-, and nano-scales play an important role in the successful acceptance of implants by the human body [44]. Surface nanostructuring is relevant to cellular response and osseointegration. On the other hand, an increase in surface roughness corresponds to a larger contact area, which in turn can stimulate bone regeneration and bonding of bone tissue to the implant [45]. The roughness of the surface significantly influences the friction coefficient of materials, where higher values lead to deterioration in the wear properties. This is also of major importance for the application of titanium and its alloys for implant manufacturing and modern biomedicine. It is also known that friction occurs between inserted implants and human bone, which can lead to the release of a large number of metal ions [50]. This is an unwanted effect as it contributes to implant failure and the occurrence of inflammatory processes in the human body. Therefore, precise control and improvement of the friction coefficient (COF) are very important to prevent these side effects. The results obtained in this work for COF show that electron-beam surface modification led to its reduction; this effect is more pronounced in the EBSM procedure with linear deflection geometry. The results obtained in this study completely correlate with these statements. The specimen characterized by the lowest surface roughness exhibited the lowest friction coefficient. However, the contact area in this case is the lowest which is expected to result in deteriorations of cell adhesion, bone regeneration, and bonding of bone tissue to the implant. Therefore, the technological conditions of the surface modification of TC4 alloys using scanning electron beams should be optimized to obtain surface architecture and topography with appropriate nano-roughness for cell adhesion and bonding of bone tissue to implants.

The possibility of employing different electron-beam geometries through the beam deflection system during the process of electron-beam surface modification allows for superb control over the process of cooling atop the surface of the specimens [28]. As mentioned, the use of different beam deflection geometries is accompanied by different cooling rates, and the resulting structure and properties of the modified materials can be very well controlled [33]. This is one of the main advantages of EBSM technology in comparison with other methods for surface modification. For that reason, the correct choice of the technological conditions of the EBSM process is very important and directly depends on the requirements for the materials and their application in the various branches of industry.

## 4. Conclusions

The impact of beam deflection geometry on the surface architecture and mechanical properties of electron-beam-modified TC4 titanium alloy have been investigated. During the EBSM process, the beam deflection geometry was in the form of a line and a circle. It was found that for linear and circular deflection geometries, the phase composition is in the form of an α’ martensitic structure. The thicknesses of the treated zones via both manners of scanning significantly differ from each other. In the case of linear geometry, it is more than 800 μm, while in the case of EBSM using circular scanning, it is 162 μm. The effect is based on different temperature evolutions due to the difference in the deposited energy density at fixed geometrical parameters. Electron-beam surface modification leads to a decrease in surface roughness to about 27 nm in EBSM with linear deflection geometry and 31 nm in circular deflection geometry, compared to that of the pure TC4 substrate (about 166 nm). The results obtained for the friction coefficient exhibit that electron-beam surface modification of the TC4 alloy leads to a decrease in the COF, with the lowest COF values obtained in the case of linear deflection geometry (0.32). The results obtained in this paper on the surface architecture and coefficient of friction would find application in the production of orthopedic and dental implants. It also provides information about the crucial role of electron-beam scanning geometry regarding surface modifications and can be used in the design of processing techniques where optimal efficiency (processed area per time) and desired surface properties can be obtained. 

## Figures and Tables

**Table 1:** Chemical composition of TC4 titanium alloy.

Element	Al	V	Fe	Co	Mo	Pd	Hf	Ti
wt. %	5.80	4.67	0.16	0.09	0.07	0.02	0.14	Bal.

**Table 2:** Surface roughness and coefficient of friction of the TC4 substrate and electron-beam-modified TC4 samples.

Samples	Surface Roughness, nm	Coefficient of Friction (COF)
substrate (pure TC4)	165.62	0.392 ± 0.008
sample 1 (line)	26.70	0.324 ± 0.009
sample 2 (circle)	31.41	0.344 ± 0.010

## Data Availability

Not applicable.
